# The unnoticed zoo: Inattentional deafness to animal sounds in music

**DOI:** 10.3758/s13414-022-02553-9

**Published:** 2022-08-25

**Authors:** Sandra Utz, Friedericke Knauss, Claus-Christian Carbon

**Affiliations:** 1 Department of General Psychology and Methodology, University of Bamberg, Markusplatz 3, 96047 Bamberg, Germany; 2 Research group EPÆG (Ergonomics, Psychological Æsthetics, Gestalt), Bamberg, Germany; 3 Bamberg Graduate School of Affective and Cognitive Sciences (BaGrACS), Bamberg, Germany

**Keywords:** Attention, Divided Attention and Inattention, Change blindness, Music cognition, Sound recognition

## Abstract

Inattentional unawareness potentially occurs in several different sensory domains but is mainly described in visual paradigms (“inattentional blindness”; e.g., Simons & Chabris, 1999, *Perception, 28,* 1059–1074). Dalton and Fraenkel (2012, *Cognition, 124,* 367–372) were introducing “inattentional deafness” by showing that participants missed by 70% a voice repeatedly saying “I’m a Gorilla” when focusing on a primary conversation. The present study expanded this finding from the acoustic domain in a multifaceted way: First, we extended the validity perspective by using 10 acoustic samples—specifically, excerpts of popular musical pieces from different music genres. Second, we used as the secondary acoustic signal animal sounds. Those sounds originate from a completely different acoustic domain and are therefore highly distinctive from the primary sound. Participants’ task was to count different musical features. Results (*N* = 37 participants) showed that the frequency of missed animal sounds was higher in participants with higher attentional focus and motivation. Additionally, attentional focus, perceptual load, and feature similarity/saliency were analyzed and did not have an influence on detecting or missing animal sounds. We could demonstrate that for 31.2% of the music plays, people did not recognize highly salient animal voices (regarding the type of acoustic source as well as the frequency spectra) when executing the primary (counting) task. This uncovered, significant effect supports the idea that inattentional deafness is even available when the unattended acoustic stimuli are highly salient.

**Supplementary Information:**

The online version contains supplementary material available at 10.3758/s13414-022-02553-9.

## Introduction

Most of us know the following social situation very well: While standing in a large crowd, looking for the next coffee shop, we overlook a waving person, even though we look directly at that personally familiar person. Since our attention is directed at something specific, which is of greater importance for us at a certain moment, we are downright blind to other aspects of that same situation. The underlying effect of situations like this is called *inattentional blindness* (first described by Neisser & Becklen, 1975; a term coined by Mack & Rock, 1998). Inattentional blindness occurs when attention is directed to a specific aspect of a visual scene, resulting in leaving us “blind” for other unattended and unexpected aspects of the same visual scene.

A well-known demonstration of this phenomenon is the Gorilla paradigm by Simons and Chabris (1999). In their study, they created 75-s long videotapes, each of which showed two teams of three players (one team wearing white and the other wearing black shirts) passing a basketball to one another. The observers were instructed either to count all passes (easy condition) or to keep a separate count of bounces and passes (hard condition) and they were also told to either pay attention to the passes of the white team or the passes of the black team. Each observer participated in only one condition of those four conditions, and 46% of the observers being occupied with counting passes between players failed to notice a person in a black gorilla costume walking through the midst of the visual scene, even though the appearance of the Gorilla is very noticeable, and, even, a bit bizarre. The appearance of the Gorilla is so startling, that when watching the scene without attending to a specific task, it is hard to believe that it is actually possible to miss it (recognition rate: 54%). We, therefore, seem to perceive and remember only those objects and details of a visual scene that receive explicit focused attention or are part of the required information to execute a given task. Accordingly, the extent of inattentional blindness seems to depend on the difficulty of the primary task because the effect occurred in more people if the difficulty of the task was higher. Observers were more likely to notice the Gorilla when attention was directed at the black team versus the white team. Thus, unexpected events seem to be recognized more likely if those events are visually similar to the ones observers are supposed to pay attention to.

Memmert (2006) investigated the role of expertise and other personality factors for inattentional blindness. He presented a basketball video similar to the one presented in the study by Simons and Chabris (1999). While a high rate of basketball nonexperts did not recognize the Gorilla (64%), basketball experts (defined as more than 5 years of experience) did much better but still missed the Gorilla in 39% of the cases. This supports the idea that expertise enables observers to direct their attention more easily toward unexpected stimuli, probably because they had more cognitive resources available to detect anything being noncentral for the game rules. However, the still high number of unrecognized occurrences of such a highly contrastive and moving Gorilla being very salient in a basketball game, demonstrates that even experts were susceptible to the effect of inattentional blindness.

Inattentional unawareness has become a widely investigated phenomenon (for a more detailed overview of studies, see e.g., Mack & Rock, 1998). Preliminary findings from the acoustic domain indicate that the underlying phenomenon is not restricted to vision only. Potentially associated phenomena have been revealed quite early (e.g., the *dichotic listening paradigm* has successfully shown that attention can be focused on one auditory stream with the result that details from the other auditory stream pass unnoticed (e.g., Cherry, 1953). The specific effect of inattentionally missing acoustic information has received little scientific attention so far. A strong influence of attention (through directed listening) to the perception of a clash of keys was revealed by Kopiez and Platz (2009). Expert and nonexpert listeners showed a significantly higher proportion of correct key error identifications when their attention was directed via instruction to possible differences in the fit between melody and accompaniment. Dalton and Fraenkel (2012) were the first to show that a noticeable auditory stimulus can be presented to both ears of an observer without detection. A binaural scene was created where a conversation between two women was presented at the same time as a different conversation between two men. Halfway through the scene, another male character “walked” through the scene and continually repeated the phrase “I’m a Gorilla” (audible for 19 seconds). Observers were instructed to either listen to the male or the female conversation. Whereas the auditory Gorilla was clearly noticeable under conditions of full attention, it was missed by 70% of observers who listened to the female conversation (i.e., a significantly higher rate than in the group that listened to the male conversation—only 10% of observers). The absence of attention can leave people actually “deaf” to a sustained and dynamic auditory stimulus that was clearly audible under normal conditions. With the given simple but effective paradigm, they confirmed the finding by Simons and Chabris (1999) that unexpected events that are more similar to the attended stream tend to be noticed more likely.

In terms of the mechanisms underlying inattentional deafness, there has been some debate on the role of perceptual load (i.e., the amount of information involved in the perceptual processing of a certain task in the manifestation of inattentional deafness). Lavie (1995) found that the perceptual load of relevant information determines selective processing of irrelevant information. In a version of the so-called Eriksen paradigm (Eriksen & Eriksen, 1974), where participants were required to indicate the identity of a target letter while a distractor was located peripherally. Irrelevant distractors were noticed only under conditions of low perceptual load and were eliminated under conditions of high perceptual load (perceptual load was operationalized through modifying the number of items among which the target appeared, i.e., either alone or among five other nontarget letters). In a follow-up study, Macdonald and Lavie (2011) confirmed the influential role of perceptual load for the perception of unexpected, task-irrelevant auditory tones (consistent with results by Lavie, 1995). Twenty-one out of 28 observers reported a brief pure tone in the low perceptual load condition, whereas only seven reported it in the high perceptual load condition. This finding, however, could not be replicated in a study by Murphy et al. (2013), who consistently failed to find such an effect across four experiments in the auditory domain. The amount of perceptual load in the attended stream did not seem to play any significant role here (see also Causse et al., 2016). The authors proposed the idea of the auditory system having spare processing capacity to process auditory information from irrelevant streams while still being able to selectively focus on only one relevant stream of sounds. They described the auditory modality as having an early-warning function that can be crucial for detecting alarm sounds in the environment that may reflect important changes, even when the perceptual demands of the task are relatively high (Murphy et al., 2013).

The high importance of investigating the underlying mechanisms of inattentional deafness is demonstrated by data from a study on air safety in the field of aeronautics. Dehais et al. (2012) let 14 pilots perform landings in a flight simulator. During the landings, an electronic alarm sound was triggered while the pilots also faced the demanding situation of a wind shear. Eight out of 14 pilots failed to detect the critical alarm, and seven of these eight pilots did not perform the adequate go-around procedure that should have followed such an alarm sound. The pilots were so focused on and occupied by reacting to the wind shear that they were deaf to the alarm sound. Inattentional deafness in air traffic can lead to perceptual errors yielding poor decision-making in cockpits and can imply lethal consequences (Dehais et al., 2012). As pilots are experts in their field, the study also demonstrated that expertise could not easily override the effects of inattentional deafness.

With regard to inattentional deafness in music, there has been little research so far. There was some research regarding a similar phenomenon, the so-called change deafness. Change deafness is the failure to notice a noticeable change, whereas inattentional deafness is characterized by a failure to notice the existence of an unexpected item. In each case, we fail to notice something that is clearly audible once we know to listen for it. In a study by Vitevitch (2003), participants had to repeat words presented via headphones. Halfway through, the speaker of the word list changed. Around 40% of participants failed to notice the change in speaker. Results furthermore showed that participants seem to fail to detect changes if they fail to focus their attention on the relevant stimulus dimension. However, performance was only tendentially slower for those who detected the change. Participants in a study by Neuhoff and Bochtler (2018) were instructed to listen carefully to a radio broadcast of sporting events to be able to answer questions regarding the broadcast afterwards. Halfway through the broadcast, the announcer changed, and 85% did not detect a change in the announcer. In a second study, participants had to listen for a change in announcer actively, and even then, 32% of participants were not able to detect a change in speakers, raising the question of the status of change deafness: Is this rather a sensitivity effect than really an attentional effect?

Other studies on change deafness could reveal that the detection of changes is limited by the cognitive capacity and therefore also by the perceptual load: The more items participants had to process, the higher the probability that they missed changes (e.g., Gregg et al., 2017). Similarly, Eramudugolla et al. (2005) showed poor change detection performance when more than four auditory objects were presented, but almost perfect performance when participants knew that a change could take place and therefore were able to focus on possible auditory changes. Consequently, change blindness seems to be dependent on perceptual load and selective attention, which are also partially responsible for the phenomenon of inattentional deafness.

Inattentional deafness to a multimodal/bimodal stimulus was demonstrated by Wayand et al. (2005). Participants watched a video similar to Simons and Chabris (1999) with two teams of players passing a basketball. There was an underlying soundtrack, and the unexpected event was a woman walking into the scene (instead of a Gorilla) and scraping her nails on a chalkboard (the sound was in the soundtrack not actually by the woman). Overall, 44% of the participants overlooked the unexpected event (both visually and aurally). Even the elimination or the strengthening of the scraping sound did not make any difference. Once participants are in a state of inattention (or focus on a different task), adding information does not decrease inattentional deafness. Koreimann et al. (2014) were the first to explicitly demonstrate inattentional deafness in music under controlled experimental conditions. One hundred fifteen participants were exposed to a music play with which they were familiar; they were also naïve to the inattentional blindness or deafness phenomenon: While listening to the first 1'50” of Richard Strauss’ *Also sprach Zarathustra* (English: *Thus Spoke Zarathustra*), participants in the experimental group were instructed to count the number of timpani beats, whereas participants in the control group were instructed to merely listen to the music piece. Actually, the music piece was modified with an inserted Electric guitar solo of 20 seconds in duration. After being exposed to the piece, participants were asked if they had noticed anything peculiar, if they heard any unfitting instruments or sounds and if they had noticed the Electric guitar solo. A study by Koelsch et al. (1999) showed that musicians are superior in preattentively processing more information out of musically relevant stimuli in comparison to nonmusicians. Koreimann et al. (2014) therefore hypothesized that musicians might be less susceptible to the effect of inattentional deafness and therefore furthermore investigated to what degree the effect is modified by musical expertise. The study showed that an unexpected musical event (the Electric guitar solo) often remained unnoticed when an explicit task (counting timpani beats) engaged attentional resources. Most participants (musicians and nonmusicians together) who were counting the timpani beats missed the Electric guitar solo (57%), whereas only a few participants in the control group, who just listened to the piece, missed it (19%). Musical training led to generally higher detection rates but was not able to fully eliminate the appearance of inattentional deafness: 52% of the nonmusicians missed the Electric guitar solo compared to 25% of the musicians (similar results were already found by Kopiez & Platz, 2009). Although the study findings by Koreimann et al. (2014) are quite indicative, the salience of the unexpected stimulus should be taken under scrutiny. Since elements of classical music in contemporary pop music or electronic remixes of well-known pieces of classical music are becoming increasingly common, an electric guitar solo in a piece of classical music might not be perceived as particularly unusual—just think of neoclassical metal music where merging classical melodies and rock music pioneered by Deep Purple (e.g., the *Concerto for Group and Orchestra* from 1969 performed by Deep Purple as a rock group and the Royal Philharmonic Orchestra as a classical orchestra). Moreover, classical themes like that from *Thus Spoke Zarathustra* often serve as soundtracks in popular movies, e.g., as in the science fiction movie *2001: A Space Odyssey* directed by Stanley Kubrick (1968). These examples make clear that many hearers are used to such pieces where the pure classical music character is lost. Consequently, it is very well possible that the electric guitar solo is a less salient stimulus than the gorilla utterance in the study by Dalton and Fraenkel (2012). Last but not least, the mere fact that Koreimann et al. (2014) made usage of just one single music play together with one specific auditory insertion at a certain time point urgently requires further replication and testing of the general effect revealed there.

## Study aims

Following these ideas, the main aim of the present study was to create an analogous experiment to the studies by Simons and Chabris (1999) and Dalton and Fraenkel (2012) in the auditory domain. More specifically, we aimed at transferring the idea of inattentional unawareness to the field of musical processing. Furthermore, the present study tried to show that a clearly audible and very salient “auditory Gorilla” can pass unnoticed by participants listening to music. Animal sounds with no connection to the music serve as unexpected stimuli and, due to their bizarreness, an actual auditory realization of a “Gorilla.” Possible individual differences between participants^1^ that influence the detection of an unexpected stimulus were also of interest. The individual ability to focus attention (measured by the deviation from the correct counting task results) on a certain task can predict the degree of susceptibility to missing unexpected targets. We hypothesized that the stronger the deviation from the correct result, the higher the probability of detecting the unexpected sound. A higher ability to focus on the task should result in missing more unexpected stimuli. Another aim of the present study was to shed light on the perceptual load debate. As demonstrated by Macdonald and Lavie (2011), a greater difficulty of the primary task (higher perceptual load) should therefore make participants in the present study more prone to miss unexpected stimuli. Finally, differences in feature similarity should impact the participants’ ability to notice the unexpected stimuli, as was proposed by Simons and Chabris (1999), so that higher feature similarity should lead to a higher chance of unexpected stimuli to be detected. In contrast to the “one-shot” design used in the studies reported above, the present study aimed at disentangling effects of music play specifics and characteristics of the interfering animal voice. Therefore, a great variety of animal voices and musical stimuli covering various music types from very different epochs was used. Also, it enables us to generalize findings from the present experiment across different genres. We hypothesize that inattentional deafness can be found regardless of unexpected sound or musical piece. To gain the data relevant for the aim of the study, musical pieces were presented in random order, with one-half of the pieces including unexpected animal sounds and the other half with the music pieces remaining original.

## Method

### Participants

The sample size of 36 was calculated a priori via a power analysis (Faul et al., 2007) based on a difference from constant (one sample case) *t* test being able to detect a medium effect size Cohen’s *d* of 0.5 (Cohen, 1988) given an α = 0.05 and a test power (1 − β) = 0.90. A total of 37 people participated in the experiment. One participant, however, had to be excluded from analysis due to interruptions during the experiment. This yields a final sample of 36 people (21 female, 15 male) ranging in age between 18 and 58 years (*M* = 32.5 years, *SD* = 12.0). Participants were mainly recruited among students of psychology from the University of Bamberg, who received course credits for their participation; the remaining participants pursued other courses of studies or were engaged in diverse occupational fields.^2^ All participants were fluent in English, which was indicated by the years they learned English at school, to ensure they would be able to adequately understand the English lyrics appearing in some of the musical pieces. All but one participant reported normal hearing, yet this one participant was not excluded because she showed no deviation in her ability to hear the animal noises in all but one of the pieces. This one piece of this one participant was then excluded from the analysis. Participants were naïve as to the purpose of the present experiment. The study was conducted according to the principles expressed in the Declaration of Helsinki and according to the ethical principles of the German Psychological Society (DFG) and the Association of German Professional Psychologists (BDP). Each participant was made aware of their right to withdraw themselves and their data from the study without consequences and without giving reasons. Written informed consent was given by each participant. The details and the rationale of the study were discussed with every participant on completion of the experiment.

### Material

Twenty pieces of music were chosen for the experiment based on two selection criteria: (1) they should possess a distinctly countable feature and (2) they should be familiar to most people (following Koreimann et al., 2014)—all music pieces are described in detail in Tables 1 and 2. The unequivocally countable feature varied between tasks concerning the lyrics (e.g., counting certain words, counting all pronouns) and tasks concerning instruments (e.g., counting bass drum beats, counting bass sounds, melodic variations, or changes). The pieces were then cut and edited (random noise was reduced) with the sound studio software Audacity 2.1.2 developed for editing audio tracks. Pieces varied in length between 21 and 84 s (*M* = 46 s; exact lengths can be retrieved from Table 1). All songs were matched in loudness with the software MP3Gain 1.3.4 to the level of 70 dB SPL (decibel sound pressure level) at maximum (note that the software is used to normalize the overall impression of how loud the song actually sounds to the human ear; the output level for the headphones was held constant across participants.). The musical pieces were then split into two groups of ten each. Ten musical pieces were modified by adding animal sounds adapted in loudness so that they were still clearly audible as being odd (an inadequate animal voice in a familiar music play) yet would not particularly stand out just on the basis of the loudness. Ten musical pieces remained original (i.e., without animal sounds) to reduce the obviousness of the aim of this study and to provide a criterion of having detected the odd sounds.

**Table 1:** Details of the musical pieces without animal sounds including musical piece, composer, genre, total duration, counting task, and total number (referring to the correct results of the counting task)

Musical piece	Composer	Genre	Total duration	Counting task	Total number
“O Fortuna” (*Carmina Burana*)	Carl Orff	Classical	24.50 s	Timpani beats	16
*Jolene*	Dolly Parton	Pop	50.69 s	Word *Jolene*	9
*Nothing Else Matters*	Metallica	Pop/ Rock	46.27 s	Bass drum beats	27
*99 Luftballons*	Nena	Pop	30.27 s	Nouns	10
The American National Anthem	A Marching Band	Classical	45.44 s	Cymbal beats	17
*Le Cygne*	Camille Saint-Saëns	Classical	60.92 s	All notes (played by the cello) that are higher than the respective note played before	27
*Macarena*	Los del Rio	Pop	33.68 s	Word *Macarena*	7
*My Generation*	The Who	Pop	29.93 s	Word *Generation*	6
The Universal Studios intro	Bryan Tyler	Classical	20.97 s	Timpani beats	11
*Geboren, um zu leben*	Unheilig	Pop	52.36 s	(German) pronouns	13

**Table 2:** Details of the musical pieces with animal sounds including musical piece, composer, genre, total duration, counting task, total number (referring to the correct result of the counting task), animal (used as added sound), animal presentation (within musical piece), and (subjective) feature similarity

Musical piece	Composer	Genre	Total duration	Counting task	Total number	Animal	Animal presentations at (s)	Feature Similarity
Symphony No. 5	Ludwig van Beethoven	Classical	29.02 s	General musical theme (four notes)	20	Gorilla	22.85; 28.40	High
Ain’t No Sunshine	Tom Jones	Pop	51.03 s	Phrase *I know*	26	Wolf	34.49; 41.49	Low
In the Hall of the Mountain King	Edvard Grieg	Classical	57.11 s	Cymbal beats	62	Cock	31.10; 43.02	High
Männer	Herbert Grönemeyer	Pop	74.88 s	Words *Mann* /*Männer*	20	Bird	36.47; 66.62	High
Hotel California	The Eagles	Pop	51.46 s	Bass tones	16	Frog	26.86; 39.94	Low
I Will Follow Him	The movie Sister Act	Pop	41.59 s	(English) pronouns	22	Dog	28.17; 37.53	High
The Moldau^a^	Bedřich Smetana	Classical	54.12 s	All notes (by the violin) that are lower than the respective note played before	23	Lion	28.91; 51.92	Low
Thus Spoke Zarathusthra	Richard Strauss	Classical	83.74 s	Timpani beats	32	Cat	38.1; 67.97	Low
The House of the Rising Sun	The Animals	Pop	43.91 s	Bass tones	30	Chickens	20.07; 36.40	Low
Cotton Eye Joe	Rednex	Pop	36.37 s	Bass tones	48	Geese	20.6; 33.12	High

^a^ excerpt starting at the first occurrence of the famous motif

Furthermore, each music play was only used once (manipulated vs. original) as a second presentation of the same base stimuli would make the participants suspicious of possible manipulations of the stimuli. Music pieces with and without animal sounds were matched regarding genre (four classical pieces, six pop songs). The music pieces were furthermore leveled in terms of the expected difficulty of the assigned counting task, so both musical pieces with and without animal sounds contained both easier and more difficult tasks (subjectively perceived difficulty revealed by a small pretest was then also additionally rated by the participants during the experiment). Each animal sound was relatively short (duration approximately between 1 and 3 s) and was inserted in two separate locations in each piece to increase the possibility of detecting it. In order to decrease the chance of detecting the animal sounds by a rigid criterion, the interval between the appearances of the two animal sounds varied, as well as their exact position within the musical pieces. In each piece, the exact position of the animal sounds was chosen, considering that the sound would neither stand alone nor be too obvious nor be masked out by a different, much louder sound. For the exact position of each of the animal sounds within each of the musical pieces, see Table 2. Animal sounds were assigned to the counting tasks concerning differences in subjectively assessed feature similarity (see Table 2). As was found by Simons and Chabris (1999), fewer participants noticed the distractor when the feature that is attended to and the distractor were dissimilar. Therefore, five counting task target sounds were assigned to animal sounds in terms of higher feature similarity (more similar in tone pitch), and five counting task target sounds were assigned to animal sounds in terms of lower feature similarity (more dissimilar in tone pitch; relatively high-pitch animal sounds assigned to low-pitch target music plays or vice versa). Lists containing all musical pieces, the corresponding tasks (assigned to each musical piece), the assigned animal sounds, as well as the level of feature similarity can be found in Tables 1 and 2.

The experiment was set up and presented on a laptop computer (Dell Latitude E6430) using the most updated Experiment Builder 1.10.1630 (SR Research Ltd., Canada) software. The musical pieces were presented via Philips SHL3060WT/00 stereo headphones (closed acoustic system with a frequency response between 10 and 22000 Hz) at a constant medium level (20% of the maximum volume), maintained constant for all participants.

### Procedure and design

**Fig. 1:**
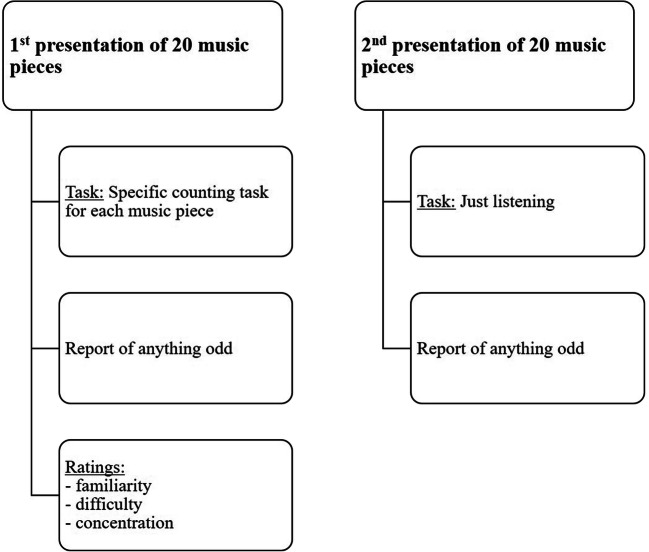
Experimental procedure

All participants were tested individually in a quiet room without audible disturbing noises. Before the experiment on the laptop started, participants were instructed to listen carefully to the musical pieces and concentrate on a task specifically set for each music piece and which always included counting certain incidents (see details in Tables 1 and 2 “Counting task” column). The participants had to focus on the respective task while counting the respective target sounds as accurately as possible. In order to motivate them and ensure they would focus their full attention on the task, they were given the prospect of winning a voucher for Amazon (worth €20) if they managed to be among the best three people taking part in the study. The delivery of all vouchers was executed right after the end of the whole experimental series. Examples of some of the counting tasks were described verbally (e.g., counting timpani beats or counting certain words of the lyrics). Participants were instructed to keep a silent mental count of the total number. The particular task was presented right before the musical piece. If the participants felt unsure about the particular target (e.g., what timpani beats sound like), they were provided the opportunity to listen to short excerpts of those targets ahead of each piece for clarification. In the first round, all 20 musical pieces were randomly presented, and after each piece, participants were asked to report the exact number they counted verbally and if they noticed anything odd. If they reported something odd, they were asked to describe it as accurately as possible and also to report how often they had perceived it. Additionally, right after the presentation, each musical piece was rated in terms of familiarity (known vs. unknown) and perceived difficulty of the counting task (7-point rating scale from 1 = *very low* to 7 = *very high*). Participants were also asked to report how concentrated they were on the task on a 7-point rating scale (from 1 = *not at all* to 7 = *very much*). After all 20 musical pieces had been presented, the sum of nontarget pieces (without animal sounds) plus all pieces including animal sounds that were not reported as containing anything odd were presented again. In this second round, participants were told to just listen to them, this time *without* attending to any counting task. After listening to those musical pieces for the second time, they were again asked to verbally report if they had perceived anything odd and describe it as precisely as possible (The experimental procedure is depicted in Fig. 1). The whole procedure lasted about 1.0 hour in total.

## Results

### Data analysis

In the following, the overall frequency of missed animal sounds, our measure for inattentional deafness, will be analyzed. We focused on several variables that are susceptible of moderating the degree of inattentional deafness: The impact of the individuals’ level of attentional focus on the frequency of missing animal sounds was investigated through analyzing their level of concentration on the task that participants rated after each musical piece, and the deviation of their counting task result from the correct number. The amount of perceptual load was measured by calculating the mean difficulty of each musical piece based on participants’ ratings (ranging from 1 = *very easy* to 7 = *very demanding*). Feature saliency between animal sounds and counting task target sounds was analyzed by four different psychoacoustical measures (loudness, specific loudness, roughness and impulsiveness) which can be used as components reflecting an inverse concept of feature *similarity*.

All musical pieces that participants indicated as being unknown to them were excluded from further analysis (i.e., 40 musical pieces in total, following Koreimann et al., 2014). This was done to ensure that being deaf to an unexpected animal sound does not result from a lack of knowledge of the musical piece and the misinterpretation of the animal sound as being part of the original musical composition.

Some participants were indeed not able to identify all of the animals correctly (especially the Lion and the Gorilla were prone to errors and were often reported as growling or snoring). We used a liberal criterion to assess an allegedly recognized odd sound as the correct animal sound in order to follow a conservative criterion to qualify missed animal sounds as inattentional deafness: We asked the participants to describe any recognized sound as accurately as possible or to reproduce it vocally and qualified everything as correctly identified as soon as the descriptions were close enough to the nature of the original sounds. Actually, we qualified all indications of additional sounds as correct recognitions in the end.

Following the inattentional blindness paradigm, animal sounds in the musical pieces were counted as missed if the participants were *not* able to report hearing the corresponding animal during the first round but were able to identify the animal sounds as odd during the second round (where the musical piece was presented once again, but this time without a counting task). Additionally, they had to confirm that the animal sound was entirely new for them, and they were sure not to have heard it during the first presentation of the piece. After listening to the respective musical piece in the second round, some participants indeed reported that they might have heard the animal sounds before; when asked why they did not report them, they stated that they interpreted the animal sounds as belonging to the musical piece. After listening to the musical piece for a second time, however, the animal sounds appeared to be more peculiar. They were therefore reported—such cases were treated as hits (i.e., correct detection of animal sounds during task performance). Furthermore, all data regarding music pieces where participants failed to manage the counting task were excluded from analysis. We cannot exclude that participants just listened to the music without executing the secondary task. However, this happened only four times across all participants (i.e., 0.6% of all cases). All these measures were implemented to estimate the degree of inattentional deafness in a very conservative way in order to effectively reduce the risk of alpha error.

### Inattentional deafness^3^

Across all participants and all musical pieces containing animal sounds, a total of 320 out of 360 musical pieces with animal sounds were included in the analysis (following our exclusion criteria mentioned above). Out of these 320, a total of 101 animal sounds were missed (no false alarms at all)—this means that 2.81 (out of 10) animal sounds per person were missed on average, one-group *t-*test against zero showed a very large effect, *t*(35) = 9.87, *p* < .001, Cohen’s *d* = 1.65, 95% CI [1.14, 2.14], according to Sawilowsky (2009). Using nonparametric testing, here Wilcoxon signed-rank test indicated a similar finding: *W* = 595.00, *p* < .001. Referring to the 320 out of 360 songs with embedded sounds which were analyzed, this means that 31.2% of all animal sounds were not detected at all. The missed animal sounds were furthermore distinguished with regard to their relative position within the experiment, as the first recognition could have changed the overall awareness and expectation of similarly odd sounds of animals within forthcoming music pieces. The following analysis controlled this: The musical piece of each participant was registered where an animal sound was recognized the first time within the experiment. All of the missed animal sounds before and after this mark were then counted separately. Sixty-six of the 102 missed animals sounds were missed before any of the other animal sounds in the experiment were detected by participants, 36 were missed after one of the previous animal sounds had already been detected. Since the total number of musical pieces included into analyses varied across participants (depending on if musical pieces were excluded due to being unknown to participants or due to a lack of managing the required task), we used the percentage of musical pieces where animal sounds were missed out of all included musical pieces to be able to compare participants among each other. Participants ranged from 0.00% to 83.33% (*M* = 32.75%, *SD* = 21.30) in their individual rate of missing the unexpected animal sounds. Analysis of the descriptive statistics of the individual musical pieces showed differences in the frequency of missing animal sounds across musical pieces ranging from 4.00% to 68.75% (descriptive statistics for all musical pieces containing animal sounds can be found in Table 3).

**Table 3:** Individual results of the musical pieces with animal sounds

Musical piece	Difficulty^a^		Deviation^b^		Missed^c^		
	*M*	*SD*	*M*	*SD*	Total^d^	Before^e^	After^f^
Ain’t No Sunshine	5.16	1.35	2.94	2.52	68.8% (22 of 32)	12	10
Cotton Eye Joe	5.91	1.36	11.50	9.47	41.2% (14 of 34)	10	4
Hotel California	4.63	1.56	0.83	1.29	13.3% (4 of 30)	4	0
Mountain King	5.97	1.20	6.72	5.03	50.0% (16 of 32)	7	9
I Will Follow Him	6.32	1.06	5.29	4.46	42.9% (12 of 28)	6	6
Männer	4.20	1.47	0.66	1.06	31.4% (11 of 35)	10	1
Symphony No. 5	5.97	1.13	6.06	4.38	8.3% (3 of 36)	1	2
Rising Sun	4.36	1.68	1.64	2.71	30.6% (11 of 36)	10	1
Moldau	6.68	0.56	8.92	4.99	4.0% (1 of 25)	0	1
Zarathusthra	5.47	1.37	5.41	5.29	21.9% (7 of 32)	5	2
Total *M*	5.47	1.27	5.00	4.12	31.24		
Total sum						65	36

^a^ rated difficulty of the task (rating from 1 = *very low* to 7 = *very high*). ^b^ deviation from the correct number in the counting task. ^c^ rate of missed animal sounds. ^d^ Total number of musical pieces is varying because unknown pieces were excluded from further analysis. ^e^ missed before one of the animal sounds was perceived for the first time. ^f^ missed after one of the animal sounds had already been perceived

### Attentional focus

A logistic regression analysis with both predictors (level of concentration on the task and the deviation of the counting task result from the correct number) was conducted for one of the musical pieces in order to predict missed/noticed animal sounds within this musical piece. The music piece *In the Hall of the Mountain King*, by Edvard Grieg, was chosen for analysis because here the number of people missing (16 of 27) and noticing (11 of 27) the animal sound was closest to equal for all presented music pieces. Therefore, the impact of factors other than those regarding inherent properties of the musical piece can best be investigated, and the two measures of individuals’ level of concentration were included in the analysis as predictors of the frequency of missed animal sounds. Only those participants who missed and those who noticed the animal sounds (all other groups were excluded) were included in the analysis. Therefore, logistic regression analysis was conducted for 27 participants. A test of the full model against a constant-only model was statistically significant, indicating that the predictors as a set reliably distinguished between participants who missed and participants who noticed the unexpected animal sound, χ^2^(2, *N* = 27) = 6.20, *p* = .045. Nagelkerke’s *R*^2^ of .277 indicated an improvement of explained variance of 27.70%. Prediction success overall was 59.30%. The Wald criterion demonstrated that only deviation from the correct number significantly contributed to prediction, *p* = .047, whereas the reported level of concentration was not a significant predictor, *p* = .219. The odds ratio value (*OR* = 1.22) indicates that when the deviation from the correct number is raised by one unit (one error in counting), the odds ratio is 1.22 times larger and therefore increases the probability of perceiving the unexpected animal sound by the factor 1.22. Making more errors in the counting task leads to a higher chance of noticing the respective animal sounds.

### Perceptual load

A simple linear regression was calculated to predict the percentage of missed animal sounds based on the perceptual load of the corresponding task. Mean ratings for task difficulty ranged from 4.20 to 6.68 (*M* = 5.53, *SD* = 0.87) between the musical pieces with animal sounds. No significant regression equation was found, *p* = .711 (i.e., the frequency of missing animal sounds could not be predicted by task difficulty).

### Feature similarity and feature saliency

To compare the frequency of missed animal sounds between musical pieces with low feature similarity (*M* = 27.70%, *SD* = 24.98) and musical pieces with high feature similarity (*M* = 35.35%, *SD* = 16.53), we conducted a paired-samples *t* test. The analysis did *not* show a statistical difference, *t*(4) = .64, *p* = .559, *n.s*. The lowest rate of inattentional deafness was found in *The Moldau*, by Bedřich Smetana (4.0%), followed by *Symphony No. 5*, by Ludwig van Beethoven (8.3%)—please refer to Table 3 for further details. Whereas feature similarity was high in *Symphony No. 5* (due to the task requiring following different instrumental voices in the piece which varied in pitch and were similar in pitch to the Gorilla) it was low in *The Moldau* (because the task required to pay attention to the violin which was consistently distant in absolute pitch from the roar of the lion).

The music piece *Ain’t No Sunshine*, by Tom Jones, with a rate of 68%, was the musical piece with the highest frequency of inattentional deafness, followed by *In the Hall of the Mountain King*, by Edvard Grieg, with a rate of 50.0%. Whereas the high howling of a Wolf and the low-frequency bass voice of the singer in *Ain’t No Sunshine* suggest a low feature similarity, the cymbals and the cock-a-doodle-doo in the *Hall of the Mountain King* were comparatively much closer in tone pitch and therefore had high feature similarity.

These subjective descriptions of feature similarities are helpful to qualitatively assess how the different animal sounds fit into the music piece. As such, they might indicate how salient the animal sounds were in the given musical context. In order to cross-check these assessments with replicable psychoacoustical measures, we analyzed all manipulated music pieces with the inserts of animal sounds and compared them with the original plays. All analyses were conducted via Head Acoustics ArtemiS SUITE © 12.0 by first identifying the maximum of both channels (left and right channels from stereo wave files) for the specific length of the animal sound duration (see Table 2) and subsequently averaging the calculated data.

We analyzed all parts of the music pieces where the animal sounds were present (vs. the respective parts of the original piece where no animal sound was available) by measuring loudness, specific loudness, roughness and impulsiveness (Table 4). We were interested in these measures because they reflect components associated with feature saliency. First of all, loudness reflects the perception of sound pressure, so it is closer to the human experience of salient stimuli. Second, roughness reflects modulation characteristics of the acoustic signal: Rough sound emissions are typically perceived as increasingly noticeable and usually also as annoying, even if the loudness remains the same. Third, impulsiveness refers to the extent of brief excursions of sound pressure, so-called acoustic impulses that significantly exceed the context sound, here the original music piece. This makes these sound qualities particularly interesting to be analyzed as potential markers for feature saliency and, thus, for the probability of detecting an animal voice in a music piece. Specifically for roughness and impulsiveness, we employed the hearing model by Sottek (1993) implemented in ArtemiS SUITE.

**Table 4:** Psychoacoustical analyses of the musical pieces (original vs. animal sounds)

Musical piece	Instance	Loudness		Specific loudness		Roughness		Impulsiveness	
		animal	original	animal	original	animal	original	animal	original
Ain’t No Sunshine	1	33.4	32.2	29.8	28.7	0.0698	0.0792	1.120	1.360
Ain’t No Sunshine	2	38.3	36.4	37.0	36.3	0.0639	0.0733	1.350	1.650
Cotton Eye Joe	1	63.4	49.7	57.6	48.0	0.0501	0.0590	0.557	0.677
Cotton Eye Joe	2	65.3	55.1	60.8	52.7	0.0471	0.0471	0.664	0.830
Hotel California	1	37.9	34.4	40.9	37.7	0.0815	0.0444	0.429	0.394
Hotel California	2	32.3	27.0	36.2	31.6	0.0843	0.0797	0.374	0.340
Mountain King	1	69.4	66.2	63.9	60.2	0.0571	0.0564	0.371	0.405
Mountain King	2	67.0	65.7	56.4	55.8	0.0597	0.0598	0.366	0.371
I Will Follow	1	58.0	57.5	56.3	53.8	0.0487	0.0698	0.485	0.455
I Will Follow	2	58.8	57.1	56.3	54.7	0.0487	0.0427	0.446	0.404
Männer	1	56.4	54.3	50.3	48.2	0.0713	0.0660	0.774	0.543
Männer	2	60.3	56.9	54.9	52.1	0.0661	0.0567	0.797	0.534
Symphony No.5	1	56.5	54.8	50.6	47.5	0.0514	0.0522	0.189	0.289
Symphony No.5	2	12.8	11.1	11.9	10.4	0.0422	0.0390	0.325	0.196
Rising Sun	1	56.3	53.9	51.7	48.9	0.0932	0.1140	0.250	0.193
Rising Sun	2	54.1	45.9	48.8	44.1	0.1150	0.1420	0.339	0.222
Moldau	1	29.7	27.5	29.1	27.5	0.0585	0.0622	0.262	0.197
Moldau	2	56.7	54.7	46.1	44.2	0.0799	0.0854	0.320	0.245
Zarathustra	1	28.9	24.4	27.9	21.8	0.0481	0.0555	0.276	0.443
Zarathustra	2	58.8	52.5	56.2	52.2	0.0644	0.0701	0.286	0.303

Instances: Part of the music piece where the animal sound was presented at first or second place. Loudness is measured as the loudness of the input signal over time in *soneGF* via loudness method according to DIN 45631; specific loudness is measured as the loudness of the input signal regarding barks. Roughness is measured as the roughness (hearing model) versus time in *asper* simulating the signal processing of human hearing and is thus capable of assessing the roughness similarly to natural human hearing. Impulsiveness was measured by impulsiveness (hearing model) versus time in the unit *iu*

To test the differences between the parts with and without animal sounds on a statistical basis, we employed two-tailed paired *t*-tests, one for each of the four employed sound qualities. For loudness as well as specific loudness, we revealed significant differences (*M*_diff_ = 3.8 and *M*_diff_ = 3.3, respectively), *t*(19) = 5.06, *p* < .0001, Cohen’s *d* = 1.13, and *t*(19) = 6.24, *p* < .0001, *d* = 1.39, respectively. Both of these differences can be qualified as large to very large effects, according to Sawilowsky (2009). For roughness and impulsiveness, no significant differences could be revealed, *t*s(19) < 1, *p*s > .3832.

**Fig. 2:**
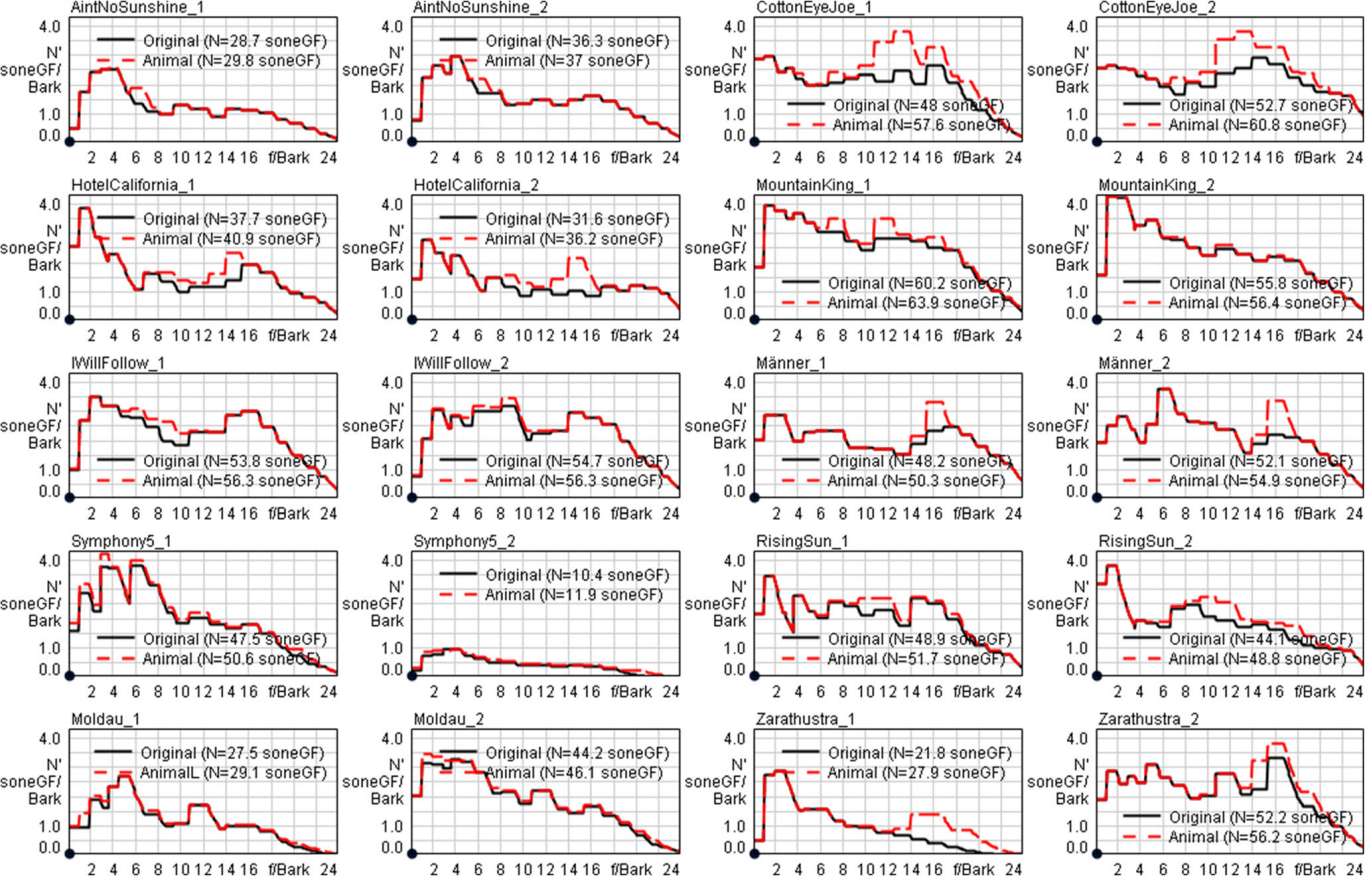
Specific loudness for the parts of the music pieces with animal voices (animal; dashed red line) versus without animal voices (original; solid black line). Loudness data is given as *soneGF* / *bark*, additionally, the integral of *soneGF* is given in the respective legends. First and second parts of the music plays (associated with the periods where animal voices appeared) always compile as pairs of diagrams labeled with “1” (first part) and “2” (second part), respectively

The specific loudness data unfolded over time for music pieces with animal sounds present vs. absent for all musical plays can be seen in Fig. 2. Diagrams of all other psychoacoustic measures can be found in the Supplementary Material (Figs. S1–S3).

**Fig. 3:**
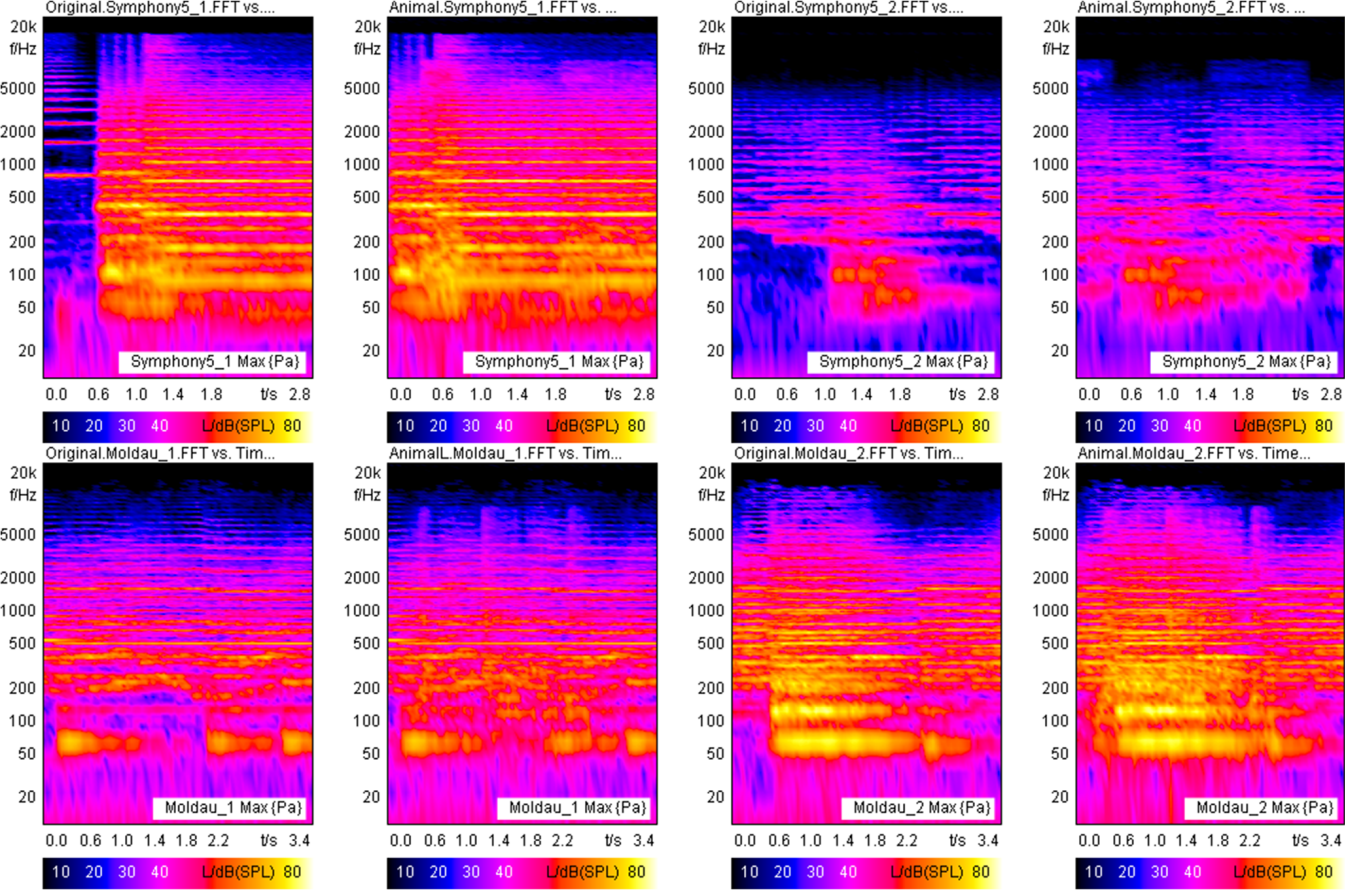
Fast Fourier transformation (FFT) versus time for the focused first and second parts of *The Moldau* with the Lion and *Symphony No. 5* with the Gorilla, respectively. First and second parts of the music plays (associated with the periods where animal voices appeared) always compile as pairs of diagrams labeled with “1” (first part) and “2” (second part), respectively. (Color figure online)

Furthermore, to give an impression of how wide the variety of different feature similarities are, we further selected two of the music pieces being affected by inattentional deafness the least and the most (i.e., *The Moldau* with the lion and *Symphony No. 5* with the Gorilla, respectively). For both musical plays, we will provide additional visualizations to see the complete unfolding of the signal of the animal sound in the musical context in terms of the involved frequencies overtime via Fast Fourier transformation (FFT). As can be retrieved from Fig. 3, the entire soundscape clearly changes for *The Moldau* when the animal voices are added. Even for *Symphony No. 5*, such a change was easily detectable via visual inspection. All other FFTs can be found in the Supplementary Material (Fig. S4).

We conducted a multiple linear regression with components of feature saliency to predict the percentage of missed animal sounds. We used the maximal absolute differences of these measures between music parts where animal voices were present and absent, so one difference value for each music play. Before conducting the regression, we analyzed the correlation pattern among all psychoacoustical measures: None of the variables except for the two loudness measures correlated with each other in a significant way, so we decided to exclude specific loudness as base measure for predicting the percentage of missed animal sounds. The multiple regression with the three independent variables loudness, roughness and impulsiveness and the dependent variable percentage of missed animal sounds was not significant, *p* = .3948. None of the independent variables could predict the dependent variable on a significant basis, *p*s > .1100. This indicates that feature saliency, at least in the limits of the here employed range, is not a valuable predictor for explaining inattentional deafness. As seen from the analyses above, other variables such as attentional focus seem to be much more promising candidates to explain how this phenomenon emerges.

## Discussion

Inattentional deafness was firstly shown by Dalton and Fraenkel (2012) were participants missed by 70% a voice repeatedly saying “I’m a Gorilla” when they were focusing on a primary conversation. In the present study, not only the validity perspective was extended by using ten excerpts of popular musical pieces from different music genres, but also acoustic signals highly distinctive from the primary sound (i.e., animal sounds) were presented. Including sounds created by nonhumans into music is not only used as artificial stimuli in experimental studies but also in experimental music—the so-called biomusic. Some famous examples are the symphonic poem “Pini di Roma,” by Respighi first performed in 1924, where a recording of a real nightingale was included in the orchestra performance, or Pink Floyd using howling and barking dogs in their songs “Seamus” (1971) and “Dogs” (1976).

Results of the present study using those biomusic elements revealed a successful transfer of the results by Simons and Chabris (1999) and by Dalton and Fraenkel (2012) to the auditory domain of musical processing. Many of the clearly audible, very salient “auditory Gorillas” with no connection to music (in contrast to Koreimann et al., 2014) passed unnoticed by participants listening to musical pieces while being occupied with an attention-consuming counting task.^4^ Importantly, this finding based on a paradigm where the primary and secondary auditory signal was very different as they stemmed from different domains (primary signal was a piece of familiar music, the secondary signal was an animal sound) essentially extends the preliminary finding of Koreimann et al. (2014) where the domain was not different between both signals.

### Attentional focus

Regression analyses showed that only the objective measure of deviation from the correct number in the counting task seems to be a significant predictor of inattentional deafness. A higher deviation from the correct answer in the counting task led to a higher likeliness to perceive the animal sounds. Therefore, more counting errors on the task led to a lower susceptibility for inattentional deafness. It could therefore be assumed that inattentional deafness is due to a lack of directing the entire attentional focus on the task. It might be that those participants with more errors generally focus less on the primary counting task and have more cognitive resources available to detect the animal sound in the first place. Another explanation might be that errors are a consequence of detecting the animal sound, diverting the attention from the primary task to the sound, and as a result, losing the count. These results are not only consistent with inattentional blindness/deafness literature (e.g., Wayand et al., 2005), but are also in accordance with findings on change deafness (see, e.g., Neuhoff, & Bochtler, 2018; tendentially in Vitevitch, 2003). However, Koreimann et al. (2014) did not find significant differences in the primary task between detecting and not detecting the unusual event. Also, the performance in the study by Vitevitch (2003) was only tendentially slower for those who detected the voice change. Future research has to look closer to the precise parameters of attentional focus on different cognitive processing levels responsible for inattentional deafness.

### Perceptual load

The frequency of inattentional deafness could not be explained by the variation of perceptual load in this study. Higher perceptual load did not induce a higher susceptibility for inattentional deafness. Considering the participants’ mean ratings of task difficulty (see Table 3), this might partially be due to the fact that tasks were generally perceived as very demanding. The mean task difficulty of 5.53 (on a rating scale ranging from 1 = *very easy* to 7 = *very demanding*) can be considered as very high, especially given the fact that the “easiest” task still received an average rating of 4.20. Therefore, the variance of task difficulty might have been too low and the tasks generally too difficult to be able to clearly differentiate high from low perceptual load.

### Feature similarity and feature saliency

The absence of an effect of feature similarity on the frequency of inattentional deafness can at least partially be attributed to a slight distinction between the two groups. Since the main aim of the present study was to show inattentional deafness to a highly striking auditory stimulus in music, attention was primarily focused on transferring the findings of the visual domain into the auditory domain by using a similar paradigm as was used by Koreimann et al. (2014). Therefore, particular emphasis was put on fitting animal sounds to the musical pieces regarding low feature similarity in order to maximize the effect. The musical pieces with particular distinct low feature similarity were then sorted into the low feature similarity condition, and the rest was sorted into the high feature similarity condition. However, the high feature similarity condition was less distinct since the fit between musical pieces and animal sounds was far more heterogeneous. The small sample size of five musical pieces in each of the groups needs to be taken into account as well, especially with regard to heterogeneity within these relatively small groups. Therefore, interpretation of the differences between musical pieces concerning feature similarity should also be conducted on an individual basis. As can be seen in Table 3, the lowest rate of inattentional deafness was found in *The Moldau*, by Bedřich Smetana, and in *Symphony No. 5*, by Ludwig van Beethoven. Whereas feature similarity was high in the *Symphony No. 5* (due to the task requiring to follow different instrumental voices in the piece which varied in pitch and were similar in pitch to the Gorilla) it was low in *The Moldau* (because the task required to pay attention to the violin which was consistently distant in absolute pitch from the roar of the lion that served as the unexpected animal sound). Yet the roar of the Lion and of the Gorilla was easily detected. A similar finding can be reported for the pieces with the highest percentage of inattentional deafness—*Ain’t No Sunshine*, by Tom Jones, and *In the Hall of the Mountain King*, by Edvard Grieg. Whereas the high howling of a Wolf and the low-frequency bass voice of the singer in *Ain’t No Sunshine* suggest a low feature similarity, the cymbals and the cock-a-doodle-doo in the *Hall of the Mountain King* were comparatively much closer in tone pitch and therefore had high feature similarity. Yet inattentional deafness had a high frequency in both pieces. Differences in feature similarity might not be sufficient to entirely explain why some musical pieces were comparatively much more prone to inattentional deafness than others. Another factor might be, that both the Lion and the Gorilla were often described as a growling sound. A growling animal can be considered as a warning sound. If a significantly lower rate of inattentional deafness could be demonstrated in those musical pieces containing an aggressive sounding animal, this would support the assumption made by Murphy et al. (2013) of the auditory modality having an early warning function which can be crucial for the detection of alarm sounds in the environment. Lastly, it should be taken into consideration that a musical piece is a very complex construct containing several different streams of musical voices. The overall complexity of the piece and the number of instruments or voices in a musical piece might have an impact and should be considered in future research regarding inattentional deafness in music.

In addition to the subjective descriptions, the feature saliency of animal sounds was further analyzed by looking at replicable psychoacoustical measures such as loudness, specific loudness, roughness, and impulsiveness. All manipulated music pieces with animal sounds were compared with the original pieces without animal sounds. All these measures were not able to predict the percentage of missed animal sounds.

## General discussion

The results of this study demonstrate that inattentional deafness in the musical realm exists, even when a highly bizarre, noticeable auditory “Gorilla” appears twice during a known musical piece. Contrary to what had previously been shown by Simons and Chabris (1999) feature similarity or our additionally used concept of feature saliency could not predict the susceptibility to inattentional deafness. One explanation might be that our measures were not the most adequate ones to capture those effects of feature similarity or feature saliency. However, as we took great care of addressing subjective as well as established objective measures, this line of argument does not seem to be very probable. Another straightforward reason could be that as soon as a certain level of perceptual load (in our case: the counting tasks) occupies our resources, we are susceptible to inattentional deafness because our attentional resources are becoming too limited. So, we will only detect the auditory “Gorillas” if our attention strays from the primary task leaving enough cognitive resources available. Indeed, we were able to document an impact of attentional focus: Whereas the subjective rating of the ability to focus attention on the specific task by the participants themselves showed no effect, the objective measure of deviance from the counting task could be found to go along with a higher frequency of inattentional deafness. A conclusion of a cause–effect relationship could not be drawn from this result, however.

Although the overall mean frequency of inattentional deafness of 31.2% may seem relatively low in comparison to findings of other authors (e.g., 57% in Koreimann et al., 2014), the results are highly remarkable. It has to be taken into account that not only did inattentional deafness appear in all but two participants and had a frequency rate of 50% or more in ten participants, but it also appeared in musical pieces that were presented after an animal sound in a previous musical piece had already been detected! More than a third (36 out of 101) of the missed animal sounds was missed after the previous detection of a different animal sound. The effect of inattentional deafness was so strong that it even appeared after participants had the chance to develop first ideas regarding the aim of the conducted experiment and to build up an expectation of more animal sounds appearing in the following musical pieces.

Whereas most of the participants expressed genuine surprise when hearing the animal sounds, because they perceived the animal sounds as very salient, other participants reported that they blended in very well with the music and could possibly be mistaken for belonging to the musical piece. This finding confirmed the decision to exclude all unknown musical pieces from further analysis to ensure that the appearance of inattentional deafness was not only due to interpreting the animal sounds as belonging to the musical piece.

Overall, very conservative and strict criteria for actual misses were applied. Animal sounds were also not counted as missed if participants reported hearing the animal sound only once after first presentation of the musical piece for the possibility that this resulted from a lack of remembering how often the animal sound was noticed rather than from inattentional deafness in one of the two appearances of animal sounds within the musical pieces. Considering these limitations in classifying inattentional deafness, the rate of approximately 31.2% is all the more impressive. Not only did inattentional deafness occur in almost all participants and all musical pieces (with very diverse rates of courses), in some participants, the phenomenon also extended over several musical pieces. To the best of the authors’ knowledge, no other study has revealed repeated susceptibility to inattentional deafness before. The likeliness of the appearance of inattentional deafness was analyzed regarding differences between people and differences between characteristics of the musical pieces. Not all findings investigated in previous studies could be transferred to the domain of inattentional deafness in music. Yet the focus of the present study lay more on investigating a possible influence of previously unattended facets and generating new hypotheses for future studies. Due to the considerably high heterogeneity of the musical pieces as well as the respective animal sounds used in this study and the high overlapping of different variables, it cannot be ruled out that parameters other than the attended variables played an important role in the frequency of inattentional deafness. Future research should attempt to identify the underlying mechanisms and musical properties that are involved in inducing or constraining inattentional deafness going beyond concepts like conceptual load or feature similarity. Special focus should be directed on the question why some individuals are especially and even repeatedly prone to the phenomenon of inattentional deafness. Individuals’ ability to concentrate and narrow attention to one task as well as differences in motivation and the eagerness to succeed, should hereby be investigated further in order to precisely identify the underlying mechanisms.

## Supplementary information

ESM 1 (DOCX 1832 kb)

## Footnotes

1 Following Koreimann et al. (2014), musical expertise should make participants less prone to missing the unexpected stimulus. With regard to our design, it is hard to find clear criteria for being an expert with regard to all the very different musical genres employing in this study.

2 Although it is difficult to find reasonable criteria for musical expertise, for a preliminary analysis of this influence on the detection performance of unexpected sounds, we used the very simple but potentially not highly discriminative criterion of more or less than 5 years of musical training (instrument and/or vocal training). We divided the group accordingly into 17 nonmusicians and 19 musicians.

3 *Musicality.* An independent-samples *t* test was conducted to compare the mean percentage of missed animal sounds across all musical pieces between nonmusicians (*M* = 36.71%, *SD* = 5.38) and musicians (*M* = 29.21%, *SD* = 4.69). With the given test power we were not able to show a significant difference between both groups, *t*(34) = 1.06, *p* = .299, *n.s*.

4 *Musicality.* In the present study, musicians and nonmusicians were equally susceptible to missing unexpected animal sounds and therefore to the phenomenon of inattentional deafness. One factor that might have contributed to this finding is the way that musicality was defined. It is hard to find experts with regard to all the very diverse musical genres used in the study. Also, the arbitrary cutoff criterion of five years to distinguish between musicians and nonmusicians was probably not ideal to distinguish between musicians and nonmusicians. Furthermore, it could be also the result of our relatively small participant group and therefore should be replicated in further studies.
