# Supplementary material for: The unnoticed zoo: Inattentional deafness to animal sounds in music
Source: Atten Percept Psychophys. 2022 Aug 25;85(4):1238–52. doi: 10.3758/s13414-022-02553-9 (PMC10167135; doi:10.3758/s13414-022-02553-9)
Supplement: Supplementary file 1 — (DOCX 1832 kb) [file 13414_2022_2553_MOESM1_ESM.docx]

**Supplementary Material**


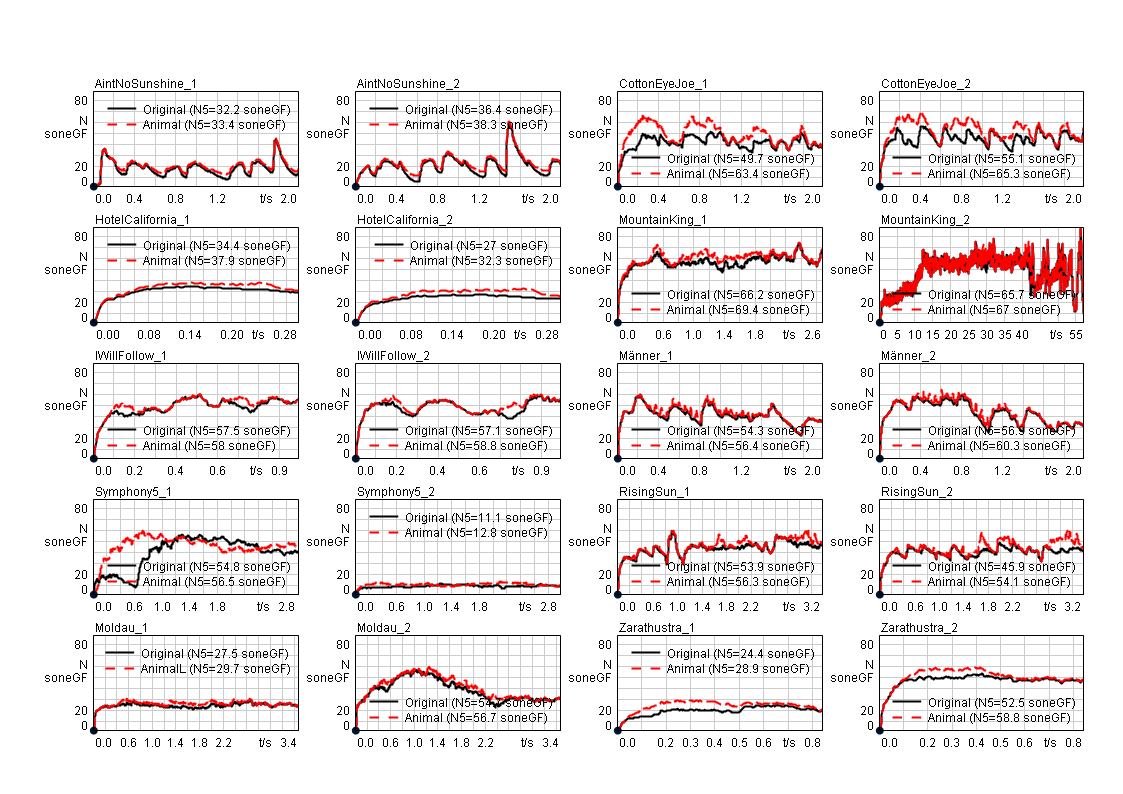


*Figure S1*. Loudness for music parts with animal voices (animal; dashed red line) vs. without animal voices (original; solid black line). Loudness is measured as loudness of the input signal over time in soneGF via loudness method according to DIN 45631; additionally the average soneGF is given in the respective legends. First and second parts of the music plays (associated with the periods where animal voices appeared) always compile as pairs of diagrams labeled with “1” (1^st^ part) and “2” (2^nd^ part), respectively.


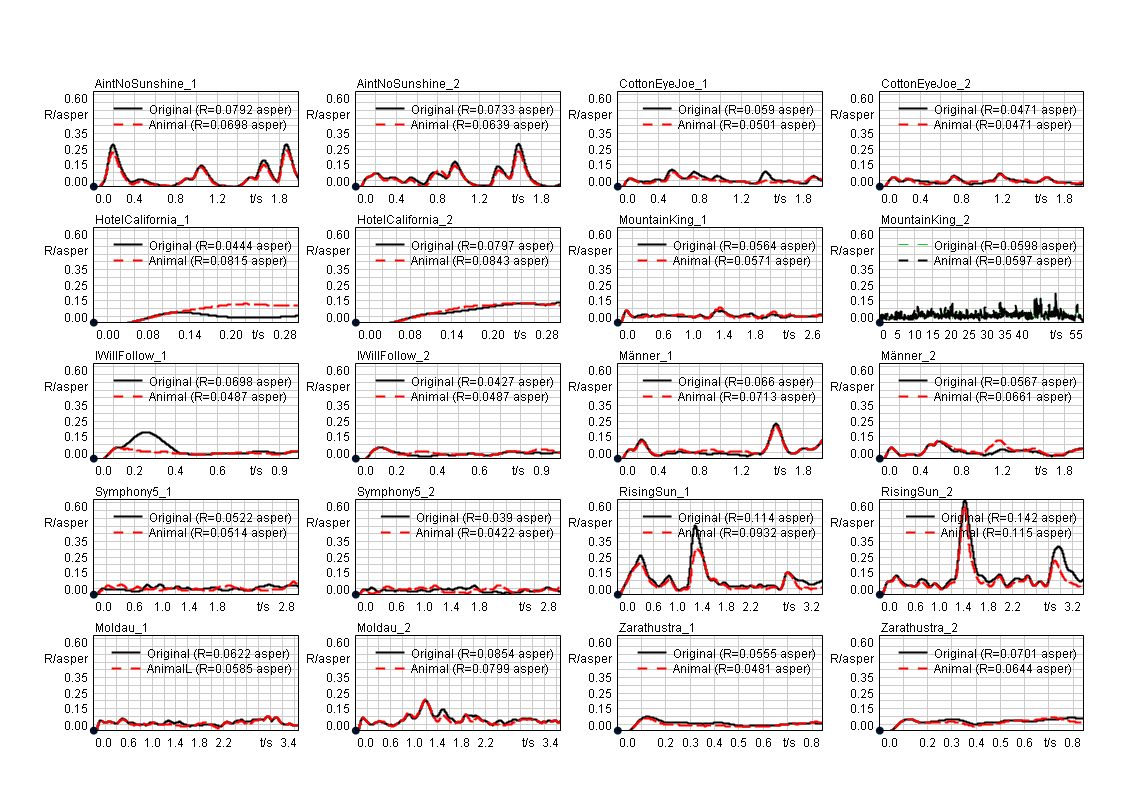


*Figure S2*. Roughness for music parts with animal voices (animal; dashed red line) vs. without animal voices (original; solid black line). Roughness is measured as roughness of the input signal over time in asper; additionally the average asper is given in the respective legends. First and second parts of the music plays (associated with the periods where animal voices appeared) always compile as pairs of diagrams labeled with “1” (1^st^ part) and “2” (2^nd^ part), respectively.


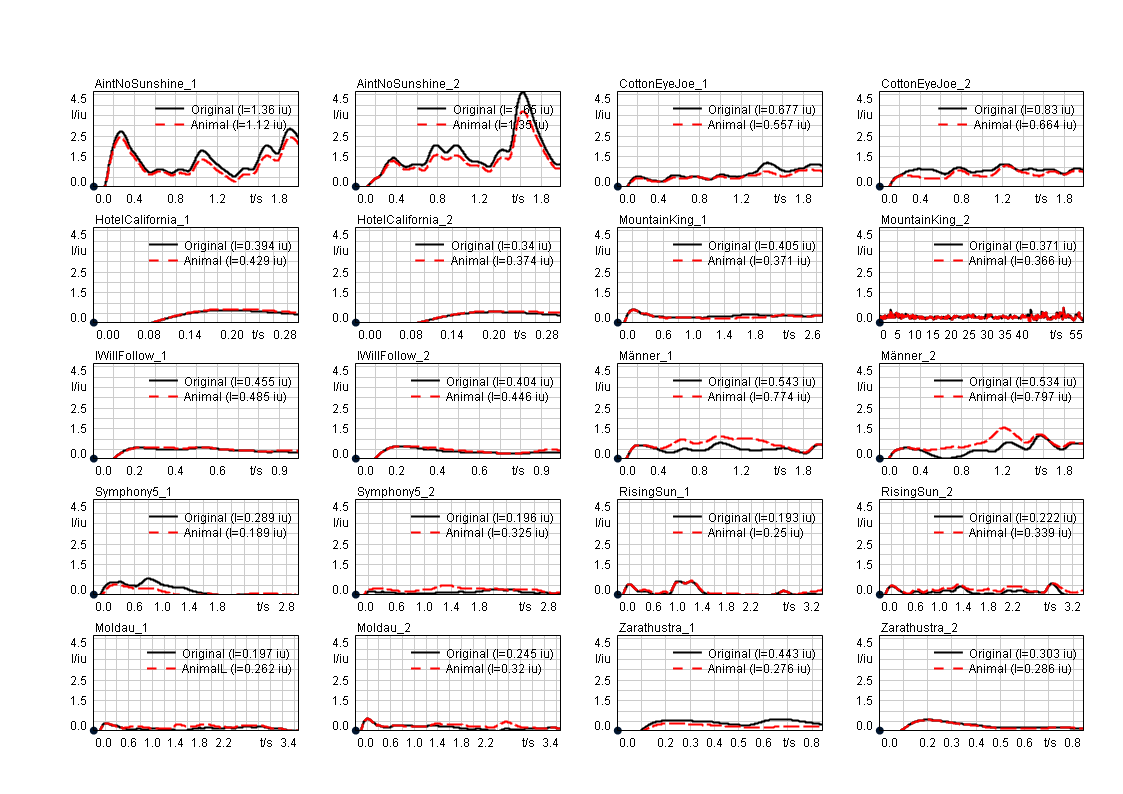


*Figure S3*. Impulsiveness for music parts with animal voices (animal; dashed red line) vs. without animal voices (original; solid black line). Impulsiveness is measured as impulsiveness of the input signal over time in ui units; additionally the average ui is given in the respective legends. First and second parts of the music plays (associated with the periods where animal voices appeared) always compile as pairs of diagrams labeled with “1” (1^st^ part) and “2” (2^nd^ part), respectively.


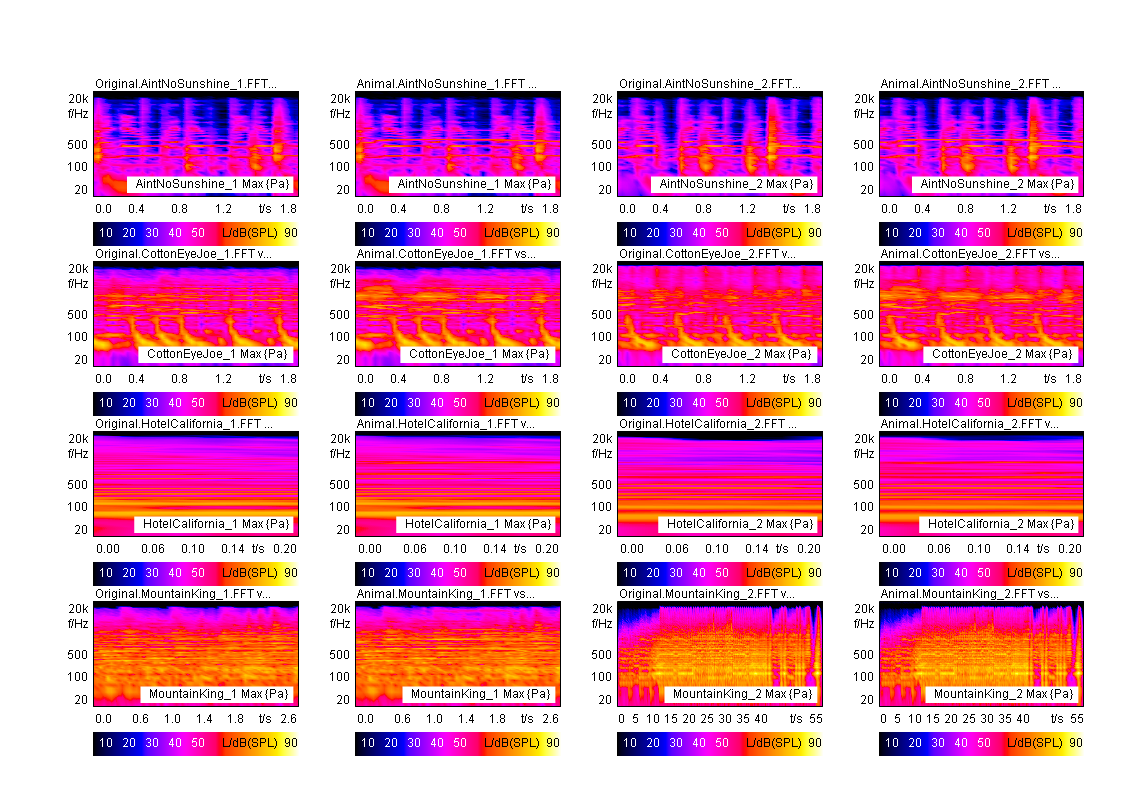


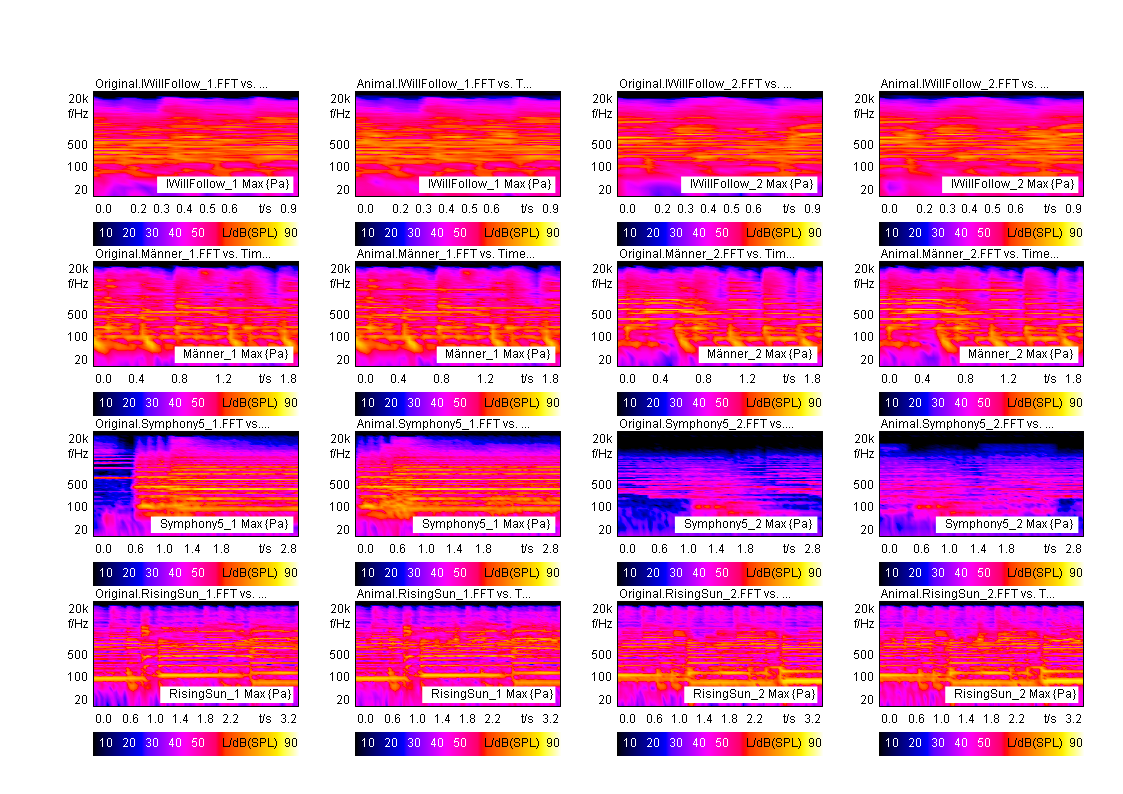


*
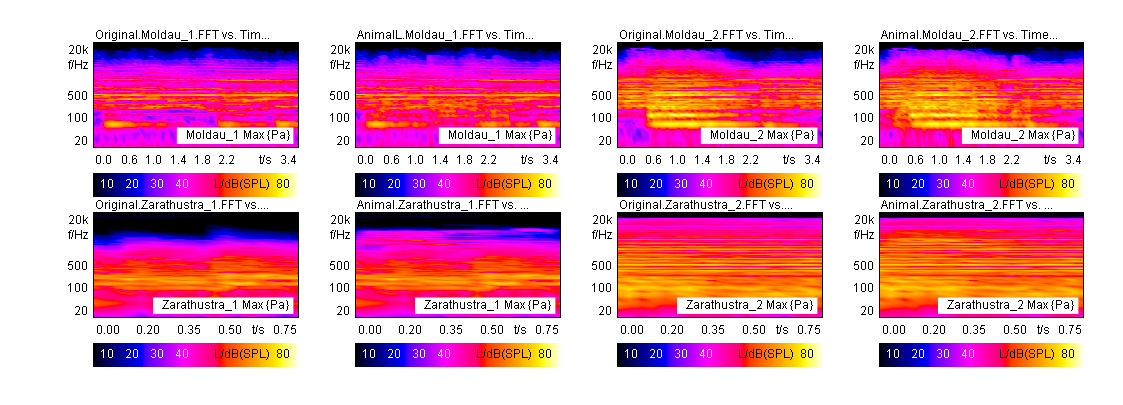
Figure S4*. Fast Fourier transformation (FFT) vs. time for the focused first and second parts of all music pieces. First and second parts of the music plays (associated with the periods where animal voices appeared) always compile as pairs of diagrams labeled with “1” (1^st^ part) and “2” (2^nd^ part), respectively.
